# Frontiers of cytokine engineering in CAR cell therapy for cancer

**DOI:** 10.3389/fonc.2025.1642022

**Published:** 2026-01-09

**Authors:** Yinghan Wu, Yan-Ruide Li

**Affiliations:** 1 Department of Microbiology, Immunology and Molecular Genetics, University of California, Los Angeles, CA, United States; 2 College of Arts and Sciences, Boston University, Boston, MA, United States

**Keywords:** antitumor capacity, cancer therapy, CAR-engineered T (CAR-T) cell therapy, chimeric antigen receptor (CAR), cytokine, immunotherapy, *in vivo* persistence, invariant natural killer T (iNKT) cell

## Abstract

Chimeric antigen receptor (CAR)-engineered T (CAR-T) cell therapy has revolutionized the treatment of hematologic malignancies, yet its efficacy in solid tumors remains limited by T cell exhaustion, restricted tumor infiltration, and an immunosuppressive tumor microenvironment (TME). Recent advances in cytokine engineering have introduced innovative strategies to overcome these barriers by modulating CAR cell survival, persistence, and cytotoxic function. This review provides a comprehensive analysis of emerging cytokine-augmented CAR platforms, highlighting mechanistic innovations such as IL-2 superkines that enhance selective CAR-T expansion, IL-15–armed CAR constructs that sustain *in vivo* persistence, and IL-12 and IL-18 co-expression systems that remodel the TME and recruit endogenous immune effectors. The roles of IL-7, IL-10, and IL-21 in preserving memory phenotypes, mitigating exhaustion, and improving metabolic fitness are also discussed in depth. Furthermore, the review explores synthetic and inducible cytokine circuits that enable spatial and temporal control of cytokine release, improving therapeutic precision and reducing systemic toxicity. Collectively, these innovations represent a paradigm shift toward next-generation, cytokine-engineered CAR therapies with enhanced efficacy, safety, and durability against both hematologic and solid tumors.

## 1 Introduction

Over the past decades, chimeric antigen receptor (CAR)-engineered cell therapy has emerged as a groundbreaking approach in the treatment of various diseases, particularly cancer and autoimmune diseases. Currently, CAR-engineered T (CAR-T) cell products targeting tumor antigens including CD19 and B cell maturation antigen (BCMA) have received the Food and Drug Administration (FDA) approval, demonstrating remarkable efficacy in treating hematological malignancies such as B cell lymphoma and multiple myeloma (MM) and achieving significant patient outcomes (1–6). Additionally, CAR-engineered natural killer (NK) cells and invariant natural killer T (iNKT) cells are being actively developed in preclinical studies, showcasing their potent tumor-killing capabilities (7–11). Despite these advancements, CAR-engineered cell therapies face several limitations, particularly in the context of solid tumors (12, 13). Challenges such as poor infiltration into tumor microenvironments (TMEs), limited *in vivo* persistence, and metabolic constraints hinder their effectiveness in treating solid malignancies (14–20).

To address these challenges, innovative strategies are being explored to enhance the efficacy and performance of CAR-engineered therapies. One promising avenue is the incorporation of cytokines-signaling molecules that play essential roles in immune modulation and cellular communication. Recent research has highlighted the potential of various cytokines, including IL-2, IL-7, IL-10, IL-12, IL-15, IL-18, and IL-21, to improve CAR-engineered cell functionality, persistence, and overall therapeutic outcomes. Each cytokine exhibits distinct biological properties; for instance, IL-15 promotes T cell proliferation and survival, while IL-12 enhances antitumor immunity by driving T cell activation and cytokine production (21–25). However, the optimal use of cytokines in CAR-engineered therapies remains an area requiring further exploration. Understanding how to effectively integrate cytokines into clinical applications is crucial for maximizing the therapeutic potential of CAR cells, especially against solid tumors where immune cell dysfunction and tumor immune evasion are prevalent.

**Figure 1:**
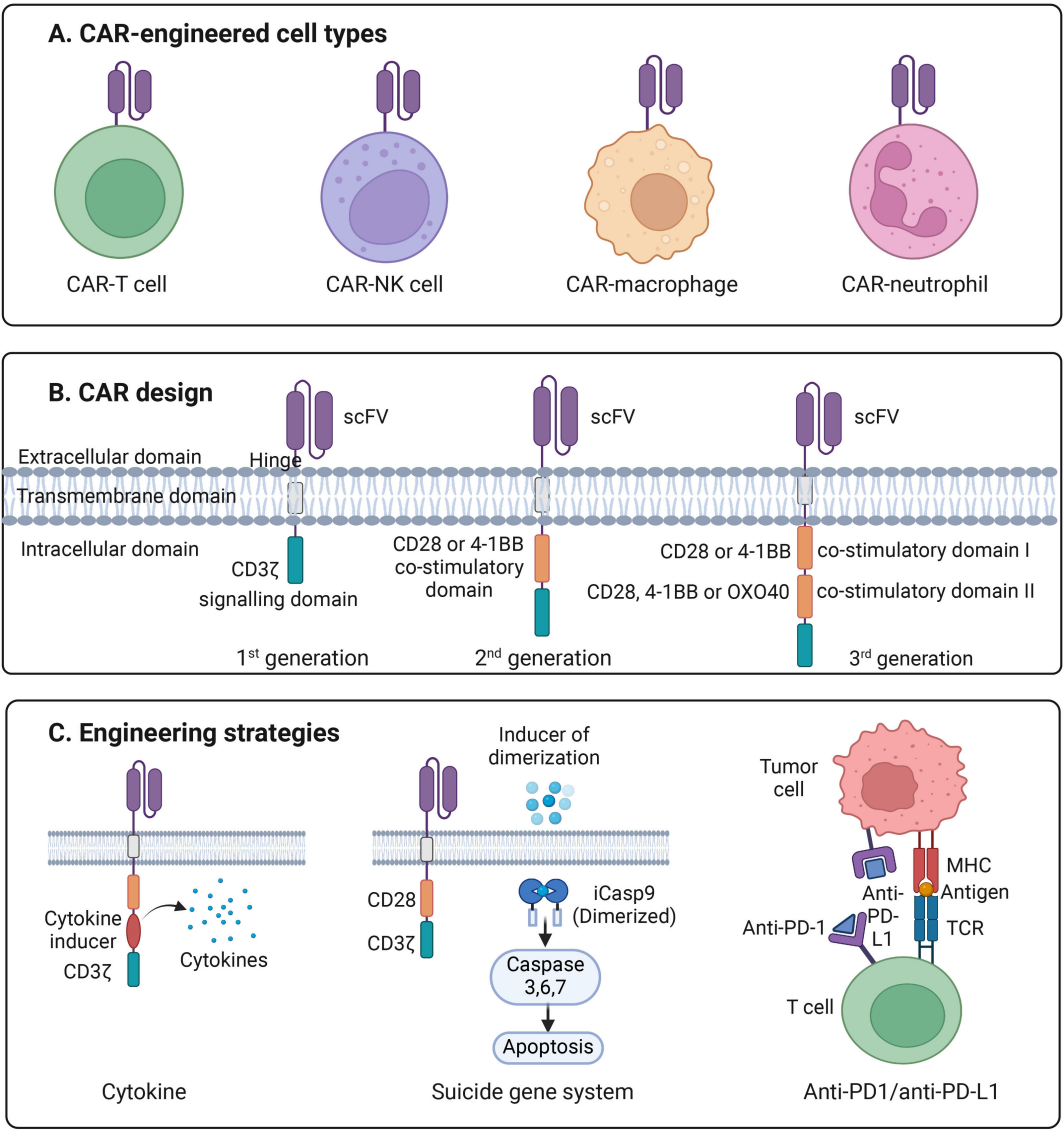
Introduction to CAR engineering. **(A)** Illustration of the four major types of CAR-engineered immune cells, including CAR-T cells, CAR–natural killer (CAR-NK) cells, CAR–macrophages, and CAR–neutrophils, each providing distinct mechanisms of antitumor activity. **(B)** Schematic representation of first-, second-, and third-generation CAR designs. Each CAR consists of an extracellular single-chain variable fragment (scFv) for antigen recognition, a hinge region, a transmembrane domain, and one or more intracellular signaling domains. First-generation CARs contain the CD3ζ signaling motif alone; second-generation CARs add a single co-stimulatory domain (CD28 or 4-1BB); and third-generation CARs incorporate two co-stimulatory domains (e.g., CD28 plus 4-1BB or OX40) to enhance activation, persistence, and cytotoxicity. **(C)** Overview of advanced CAR-engineering strategies, including cytokine engineering to augment proliferation and survival, incorporation of a suicide gene system such as inducible caspase-9 (iCasp9) to allow controlled apoptosis, and checkpoint blockade via anti-PD-1/anti-PD-L1 modules to overcome T-cell exhaustion and suppress inhibitory signaling within the tumor microenvironment.

In this review, we summarize the current advancements in cytokine engineering within CAR cell therapy, discussing the various cytokines employed, their mechanisms of action, and the challenges associated with their clinical application (**Table 1**, **Figure 1**). We propose novel strategies for optimizing cytokine use in CAR-engineered therapies, aiming to enhance the therapy effectiveness and broaden the applicability in cancer treatment. Through the comprehensive summary, we provide insights that will guide future preclinical research and clinical practices in the field of CAR cell therapy.

**Table 1:** Table comparing key cytokines used or considered for cytokine-engineered CAR cell therapies.

Cytokine	Receptor (major chains)	Effect on CAR cells (proliferation, persistence, exhaustion)	Typical CAR delivery formats	Main risks and toxicity
IL-2	IL-2Rα (CD25) + IL-2Rβ + γc	Strong driver of proliferation and effector differentiation; can drive terminal differentiation and expand Tregs; possible functional exhaustion and mixed effects on durability.	Systemic IL-2 dosing; CAR co-expression (secreted and cis-tethered); engineered or orthogonal IL-2 variants to restrict signaling.	High systemic toxicity (capillary-leak, hypotension), non-selective bystander activation including Tregs; exacerbates CRS/ICANS risk.
IL-7	IL-7Rα (CD127) + γc	Supports T cell survival and homeostatic proliferation of less-differentiated cells; favors persistence and stem-like memory; may reduce terminal exhaustion.	Membrane-bound/tethered IL-7, CAR co-expression or *ex vivo* culture supplementation.	Generally lower systemic toxicity profile compared with IL-2; risks arise if systemic high exposure causes lymphoproliferation or off-target effects.
IL-10	IL-10Rα + IL-10Rβ	Anti-inflammatory; can reduce exhaustion and support mitochondrial fitness in CAR-T cells	CAR transgene (secreted)	Potential immunosuppressive activity on bystander immunity if systemic; theoretical risk of blunting some antitumor immune responses if unconfined.
IL-12	IL-12Rβ1 + IL-12Rβ2	Strongly pro-inflammatory; reprograms TME, boosts IFN-γ and endogenous immunity; can increase effector function but risk of systemic inflammation.	CAR transgene (secreted IL-12 or local delivery), tumor-localized expression strategies.	Potent systemic inflammation, severe toxicities historically with systemic IL-12; localized expression designed to limit systemic exposure.
IL-15	IL-15Rα (presentation) + IL-2/15Rβ + γc	Promotes memory/stem-like CD8^+^ phenotype, persistence and homeostatic proliferation with less Treg expansion than IL-2; improves durability.	CAR transgene (secreted, membrane-bound/tethered), inducible expression, or co-delivery.	Can increase CAR expansion and CRS incidence; potential for uncontrolled proliferation if not regulated; motivates inducible/tethered/suicide-gene pairing.
IL-18	IL-18Rα + IL-18Rβ	Enhances IFN-γ production, boosts innate activation and T cell function; can revive dysfunctional and exhausted CAR-T cells.	CAR transgene (secreted).	Pro-inflammatory effects could increase CRS/immune-mediated toxicity; but early reports suggest tolerable safety when delivered in CAR-restricted approach.
IL-21	IL-21R + γc	Promotes less-differentiated phenotype, enhances cytotoxicity, as well as transduction and expansion quality in manufacturing; can preserve stem-like T cells and reduce exhaustion.	*Ex vivo* culture supplement; CAR constructs co-expressing IL-21.	Lower classical vascular toxicity than IL-2; concerns focus on effects on differentiation balance and possible impact on autoimmunity if systemic.

## 2 CAR designs

### 2.1 Extracellular antigen binding domain

The antigen-binding domain is a critical component of CARs, comprising a single-chain variable fragment (scFv) derived from the variable heavy (VH) and variable light (VL) chains of antibodies. This domain is essential for enabling CAR-T cells to specifically recognize and bind to tumor-associated antigens (TAAs) on cancer cells, facilitating MHC-independent T cell activation.

One of the key considerations in designing the antigen-binding domain is the optimization of binding affinity. The scFv must have sufficient affinity to ensure effective recognition and activation of CAR-T cells, but not so high that it induces activation-induced cell death (AICD) or causes toxicity (26). The structural arrangement of complementary determining regions (CDRs) plays a significant role in fine-tuning this affinity to balance efficacy and safety (27).

Additionally, the characteristics of the targeted epitope, such as its location and density on tumor cells, are critical. An ideal epitope is one that is highly accessible and present in adequate density to promote effective CAR-T cell engagement without triggering excessively strong or off-target signaling. These factors together contribute to the functional performance of CAR-T cell therapies in targeting cancer cells.

### 2.2 Extracellular hinge domain

The hinge region in CARs is an extracellular segment that connects the transmembrane domain to the scFv. One of the key roles of the hinge is to provide structural flexibility, supporting accessibility of target epitopes for the CAR. A well-designed hinge enhances the CAR’s ability to form stable interactions with target cells while maintaining sufficient mobility to navigate the complexities of the TME.

In terms of the functionality of CAR-T cell, variations in the hinge’s composition can alter the CAR-T cell’s performance ranging from CAR expression to signal transduction. Additionally, the length of the hinge plays a critical role in maintaining optimal distances between CAR-T cells and target cells, promoting efficient immunological synapse formation, which is essential for successful CAR activation and tumor cell destruction (28).

Hinge regions are commonly derived from proteins such as CD8, CD28, or IgG. However, IgG-derived hinges can interact with Fc receptors, potentially leading to CAR-T cell exhaustion and reduced persistence *in vivo*. Thus, selecting the appropriate hinge composition is crucial for optimizing CAR-T cell performance in therapeutic applications.

### 2.3 Transmembrane domain

The transmembrane domain of CARs serves a dual purpose: it anchors the CAR within the T cell membrane and plays a critical role in influencing CAR functionality.

Beyond its structural role, the transmembrane domain can affect the expression and stability of the CAR on the T cell surface. For example, the CD3ζ transmembrane domain promotes CAR dimerization and facilitates its incorporation into the TCR complex. This enhances T cell activation, although it may compromise the stability of the CAR on the cell surface. The stability of the CAR is a crucial factor in determining its longevity and effectiveness in therapeutic applications.

The transmembrane domain also influences cytokine production and sensitivity to AICD. For instance, CAR-T cells with a CD8α transmembrane domain tend to produce lower levels of pro-inflammatory cytokines compared to those with a CD28 transmembrane domain (29). This difference in cytokine production can impact the overall efficacy and durability of CAR-T cell responses, with CD28-based designs potentially offering enhanced activation at the cost of higher inflammatory activity. Thus, the choice of transmembrane domain is a key factor in optimizing CAR functionality and therapeutic outcomes.

### 2.4 Intracellular signaling domain

The intracellular signaling domain plays a crucial role in transmitting activation signals once the CAR engages its target antigen. First-generation CARs were designed with only the CD3ζ signaling domain, but these proved insufficient for generating durable therapeutic responses in clinical settings (30). Second-generation CARs introduced a single co-stimulatory domain, such as CD28 or 4-1BB, in combination with CD3ζ (31–34). This advancement significantly improved therapeutic efficacy, particularly in hematological malignancies, and is now being investigated for use in solid tumors. Third-generation CARs further enhance activation by incorporating two co-stimulatory domains alongside CD3ζ. Typically, the first co-stimulatory domain is either CD28 or 4-1BB, while the second can be CD28, 4-1BB, or OX40. This combination is designed to amplify T cell activation and persistence. However, preclinical results for third-generation CARs have been mixed, underscoring the complexities and challenges in optimizing these designs for clinical application.

### 2.5 Unique CAR designs for CAR-NK cell products

NK cells play a vital role in the body’s anti-tumor defense, and their anti-tumor efficacy can be significantly enhanced through engineering with CARs. One promising approach involves incorporating the NKG2D receptor, an activating receptor on NK cells that detects stress-induced ligands commonly expressed on tumor cells (35). By integrating NKG2D into CAR constructs, researchers can harness the inherent cytotoxic abilities of NK cells while equipping them with a more targeted mechanism to identify and eliminate cancer cells (36).

The transmembrane domain of NKG2D plays a crucial role in ensuring the receptor’s integration into the NK cell membrane, thereby maintaining the CAR’s structural integrity and facilitating proper signaling upon ligand binding. This domain is essential for the functionality of the CAR, allowing the NK cells to effectively recognize tumor cells and initiate an immune response.

Furthermore, the intracellular signaling domain of the NKG2D-based CAR is often combined with co-stimulatory molecules such as 2B4 or CD3ζ, which enhances the signaling pathways triggered by antigen recognition (37). This combination not only amplifies cytotoxic activity but also promotes cytokine production, which strengthens the overall anti-tumor response. By incorporating NKG2D and optimized signaling domains, CAR-engineered NK (CAR-NK) cells are equipped to provide a more potent and sustained attack against cancer cells.

## 3 Cytokines incorporated into the CAR-engineered cells

### 3.1 IL-2

Interleukin-2 (IL-2) is a pleiotropic cytokine critical for the proliferation and differentiation of T cells, playing a central role in adaptive immunity. Encoded by the IL-2 gene located on chromosome 4, IL-2 is primarily produced by activated CD4^+^ T cells and shares the common cytokine receptor γ chain also seen in IL-4, IL-7, IL-9, IL-15, and IL-21 (38). IL-2 functions by binding to its receptor complex, composed of IL-2Rα, IL-2Rβ, and the common γ-chain, activating signaling pathways such as JAK/STAT to initiate differentiation of T cells and promoting their expansion (39).

The structure of IL-2 follows a short chain type I cytokine composed of four alpha-helical bundle “up-up-down-down” configuration, this helical structure allows for specific binding with two classes of receptors: immediate affinity receptors, which contain IL-2Rβ and γ_c,_ and high-affinity receptors which contains IL-2Rα, IL-2Rβ, and γ_c_ (40). In the situation of resting lymphocytes and NK cells, IL-2 signals via the intermediate affinity receptors. On the other hand, activated lymphocytes causes an increase in low and high-affinity receptors due to additional expressions of IL-2Rα. In terms of IL-2Rβ, it is expressed on many immune cells at baseline without certain activation, however, T cell receptor and IL-2 stimulation also induces expression (41). Additionally, NK cells and memory T cells express higher levels of IL-2Rβ compared to other immune cells, making them more responsive to IL-2 signaling.

There are three main signaling pathways activated by IL-2, including the JAK-STAT pathway, the RAX-MAP kinase pathway, and the PI 3-kinase/AKT pathway (42). When IL-2 binds with IL-2Rα, dimerization of IL-2Rβ and γc follows which results in JAK kinase activation, leading to phosphorylation of IL-2Rβ and creations of docking sites for STAT5A and STAT5B proteins, which are transported to the nucleus to drive cell differentiation, survival, and target gene expression. Depending on the specific tyrosine phosphorylation in IL-2Rβ, IL-2 signaling also activates RAS-MAP kinase pathway which modulates cell growth and differentiation. For example, the phosphorylation of Y341 residue allows recruitment of SHC1, which is subsequently phosphorylated to recruit GRB2 and SOS to support GTP loading of Ras and activation of MAP kinase cascade. Lastly, the tyrosine phosphorylation of Y395 in IL-2Rβ causes recruitment of regulatory subunit p85 of PI3K to the IL-2 receptor. PI3K generate lipid second messenger such as PI(3,4,5)P3 which recruits proteins including PDK1 and AKT to the plasma membrane. PDK1 then phosphorylates AKT, and the activated AKT then promotes effector T cell differentiation (40).

Other than its signal transduction pathways, IL-2 has also been shown to effectively reduce tumor growth. In one study, researchers engineered the wild-type IL-2 to create a superkine variant that binds to the IL-2Rβ and γ_c_ at higher affinity. This superkine is then linked with IL-33 genes via a T2A self-cleaving peptide and inserted into a retroviral vector alongside the CAR construct (TA99 CAR) targeting tumor-associated antigens. To test the efficacy of cytokine-engineered CAR-T cells, C57BL/6 mice were inoculated with B16F10 melanoma cells, after tumors were established for 6 days, mice were i.v. administered TA99 CAR-T cells (not cytokine-engineered), and the results showed no effect on tumor growth compared to nontreated mice (43). However, TA99 CAR-T cells expressing both Super2 and IL-33 (Super2 + IL-33) was shown to have enduring effectiveness in delaying medium-sized tumor growth (∼7.5 mm × 6 mm) when administered on day 11 after inoculation. However, a delay in Super2 + IL-33 treatment results in variable responses against relatively larger tumors (∼10.5 mm × 8 mm) suggesting for engineering strategies involving Super2 in combination with other interleukins (43).

The administration of IL-2 to boost CAR-T cell expansion and persistence has clear theoretical appeal, as higher *in vivo* expansion of CAR-T cells correlates with better responses. Indeed, early serum IL-2 levels post infusion have emerged as a reliable biomarker of CAR-T expansion in large patient cohorts. However, the clinical adaptation of IL-2 boosting faces significant toxicity and selectivity challenges. High-dose systemic IL-2 historically induces severe adverse events including capillary-leak syndrome, hypotension and multi-organ dysfunction, and its non-selective activation of endogenous immune cells (including Tregs) may blunt efficacy or exacerbate toxicity (44, 45). Within CAR-T platforms, this translates into increased risk of severe CRS/ICANS or off-target immune activation. To circumvent these risks, engineering strategies such as orthogonal IL-2/IL-2R pairs, CAR-T-specific IL-2 fusion molecules, or tethered/locally restricted IL-2 delivery are under preclinical evaluation and show promise in enhancing CAR-T efficacy while limiting systemic toxicity (46). Nevertheless, to date few human trials have evaluated IL-2-augmented CAR-T, and the balance of enhanced anti-tumor effect versus added immune toxicity remains to be fully characterized. Future clinical translation will depend on carefully optimized dosing, timing, and cytokine selectivity within the CAR-T product design.

### 3.2 IL-10

Interleukin-10 (IL-10) is a cytokine well-known for its potent anti-inflammatory effects, playing a crucial role in modulating the immune response to pathogens and maintaining cellular homeostasis. Encoded by the *IL-10* gene located on chromosome 1, IL-10 is primarily produced by regulatory T cells, macrophages, and monocytic myeloid cells. Its synthesis can be triggered by inflammatory cytokines or tissue damage, allowing it to function in areas of chronic inflammation, including fibroblasts, cytotoxic T cells, and tumor cells (47).

Structurally, IL-10 belongs to the class II cytokine family and exists as a soluble 36 kDa homodimer, composed of two monomers featuring a unique six alpha-helix structure, stabilized by disulfide bonds (48). The activity of IL-10 is initiated when its homodimer binds to its receptor, composed of two subunits: IL-10R-alpha (IL-10RA), which is primarily expressed on immune cells, and IL-10R-beta (IL-10RB), which is found on various cell types. Upon IL-10 binding to IL-10RA, a signaling cascade is triggered, leading to the phosphorylation of Jak1 and Tyk2 kinases, which in turn activates the transcription factor STAT3.

Once activated, STAT3 translocate into the nucleus, where it binds to specific DNA sequences in the promoters of target genes, inducing their expression. This results in the production of suppressor of cytokine signaling 3 (SOCS-3), which inhibits inflammatory responses by blocking the MAPK and NF-κB pathways, thereby reducing the expression of pro-inflammatory genes. STAT3 activation also stimulates the production of the IL-1 receptor antagonist (IL-1RN), a decoy protein that prevents IL-1β from binding to its receptor and initiating pro-inflammatory signaling. In addition to STAT3, IL-10 signaling also activates other transcription factors, such as STAT1 and STAT5, which enhance the function of regulatory T cells, helping to further control excessive inflammation.

Beyond its well-characterized anti-inflammatory properties, IL-10 has also been shown to improve anti-tumor efficacy in the context of CAR-T cell therapy. In one study, mouse IL-10 was linked to a second-generation anti-HER2 CAR using a cleavable 2A peptide sequence, and human IL-10 was similarly linked to a second-generation anti-CD19 CAR. The anti-tumor efficacy of these engineered CAR-T cells was evaluated in a mouse model of MC38-HER2 colon adenocarcinoma, where the tumor microenvironment was established through tumor inoculation followed by sublethal lymphodepletion via irradiation (49).

The CAR-T cells engineered to express IL-10 demonstrated significantly enhanced proliferation and effector functions compared to standard CAR-T cells. In mouse models, these IL-10-modified CAR-T cells successfully eradicated solid tumors, including those expressing HER2 and CD19. Additionally, the study showed that IL-10 expression preserved mitochondrial structure and increased oxidative phosphorylation in the CAR-T cells, leading to enhanced tumor-killing capacity (49). This research underscores the potential of IL-10-modified CAR-T cells in improving anti-tumor responses, particularly in challenging TME.

### 3.3 IL-12

Interleukin-12 (IL-12) is a proinflammatory cytokine that plays a crucial role in activating NK cells, inducing IFN-γ production, and generating lymphokine-activated killer cells, making it a key regulator of host immunity (50). The IL-12 family includes other cytokines, such as IL-23, IL-27, and IL-35, which share structural similarities but have distinct biological activities. While IL-12 and IL-23 act as positive regulators and are considered pro-stimulatory cytokines, IL-27 and IL-35 function as negative regulators with immunosuppressive roles (51).

Structurally, IL-12 is a heterodimer consisting of a 35-kDa (p35) light chain and a 40-kDa (p40) heavy chain, covalently bonded together. The genes encoding these subunits are located on separate chromosomes in humans, with p35 on chromosome 5q31–33 and p40 on 3p12–q13.2 (52). IL-12 is distinct as a cytokine because it functions in association with a soluble form of its receptor. The p35 subunit resembles single-chain cytokines, while the p40 subunit shares similarities with the extracellular region of the hematopoietic cytokine receptor family. Additionally, IL-12 has structural features in common with the IL-6 cytokine family, but unlike IL-6, p35 is secreted only when combined with the p40 subunit, making IL-12 a specialized cytokine complex within its family (53).

The signal transduction pathway of IL-12 begins when its two subunits bind to the IL-12 receptor, which consists of two chains: IL-12Rβ1 and IL-12Rβ2. Upon binding, IL-12Rβ1 recruits tyrosine kinase 2 (TYK2), a member of the JAK family, while IL-12Rβ2 phosphorylates JAK2. This leads to further phosphorylation of a tyrosine residue on the associated receptor subunit, allowing STAT proteins to interact with IL-12Rβ2. STATs, which are then activated by JAK, translocate to the nucleus to regulate gene transcription, either promoting or repressing specific gene activity (54).

In CAR-engineered cell therapy, engineering IL-12 into CAR cells has demonstrated improvements in immune response and tumor-fighting capabilities. In one study, human iNKT cells were transduced using a gamma retroviral vector encoding the p40 and p35 subunits of IL-12, linked to GFP, and then co-cultured with CD19^+^ tumor cells to assess antitumor efficacy. The results showed that IL-12-engineered CAR-iNKT cells exhibited prolonged antitumor activity and persistence. These engineered cells also demonstrated enhanced polyfunctionality, superior expansion, and sustained high levels of CD62L expression *in vivo*. In a mouse model, NSG mice implanted with the BV-173 leukemia cell line further demonstrated the long-lasting effectiveness of IL-12-engineered CAR-iNKT cells (55).

### 3.4 IL-15

Interleukin-15 (IL-15) is a pleiotropic cytokine closely related to IL-2, belonging to the 14- to 15-kDa class of the 4-α helix bundle family. The gene encoding IL-15 is located on chromosome 4, and the cytokine is widely produced by activated myeloid cells, fibroblasts, and epithelial cells (56). IL-15 has been recognized for its critical role in the development and activation of several immune cell types, including CD8^+^ T cells, NK cells, iNKT cells, gamma delta (γδ) T, and mucosal-associated invariant T (MAIT) cells (57–63). IL-15 also induces activated B cells to produce antibodies, thereby supporting both innate and adaptive immune responses, making it an effective immunomodulator in combating pathogens.

Structurally, IL-15 is a glycoprotein characterized by its antiparallel 4-helix bundle, arranged in an up-up-down-down helical pattern typical of short-chain helical cytokines. The protein contains two disulfide bridges that stabilize its interaction with its receptor complex, ensuring efficient signaling (64). IL-15 exerts its biological activity by binding to a receptor complex composed of IL-15 receptor alpha (IL-15Rα), the beta subunit (IL-2R/15Rβ), and the common gamma chain (γc). This binding can occur either in cis-presentation, where IL-15 binds to IL-15Rα and IL-2R/15Rβ on the same cell, or in trans-presentation, where the IL-15/IL-15Rα complex is presented to a different cell expressing the IL-2R/15Rβγc transducing receptors.

Upon receptor binding, Janus kinases (JAK1 and JAK3) are activated, leading to the phosphorylation of tyrosine residues on the receptor and the subsequent activation of STAT proteins, such as STAT3 and STAT5. These phosphorylated STAT proteins dimerize and translocate to the nucleus, where they activate genes responsible for enhancing cell survival and proliferation. In parallel, IL-15 signaling activates the phosphatidylinositol 3-kinase (PI3K)/AKT pathway. IL-15 binding recruits PI3K, which interacts with lipid targets on the cell membrane, increasing phosphatidylinositol-3,4,5-triphosphate (PI(3,4,5)P3) levels. This triggers the activation of PDK1, which phosphorylates AKT at the Thr308 position, leading to downstream signaling events crucial for cell expansion, metabolic regulation, and cytotoxic functions (65).

In the context of CAR engineered cell therapy, IL-15 plays an essential role in enhancing the effectiveness of the therapeutic cells. For example, a second-generation anti-CD19 CAR was combined with human IL-15 and an inducible caspase-9 suicide gene using a cleavable 2A peptide sequence. This combination promotes T cell expansion and survival, sustains IL-15 production following stimulation, and improves antitumor activity against CD19^+^ tumors while reducing T cell exhaustion (66). In a recent clinical trial evaluating IL-15–engineered GPC3-targeted CAR-T cells for the treatment of hepatocellular carcinoma (HCC), IL-15 expression markedly enhanced CAR-T cell persistence, proliferation, and antitumor efficacy *in vivo*, demonstrating the potential of cytokine-armored CAR constructs to overcome the immunosuppressive tumor microenvironment (22). However, this enhanced activity was accompanied by severe treatment-related toxicities, particularly cytokine release syndrome (CRS), underscoring the need for optimized IL-15 regulation strategies, such as inducible or membrane-bound IL-15 expression, to balance therapeutic potency with safety (22, 23).

In another approach, cryopreserved human cord blood-derived CD34^+^ hematopoietic stem and progenitor cells (HSPCs) were transduced with a lentivector delivering a human iNKT TCR, a designated CAR, and additional IL-15 genes (67–69). The resulting ^Allo/15^BCAR-NKT cells exhibited robust proliferation and produced high levels of Th1 cytokines, including IFN-γ and TNF-α. Within the TME, these IL-15-enhanced cells demonstrated superior efficacy in eliminating primary multiple myeloma (MM) cells compared to conventional CAR-T cells. In preclinical models, these engineered iNKT cells led to complete tumor elimination in nine out of ten mice, with all surviving for more than 80 days. Importantly, this treatment demonstrated a favorable safety profile, with no signs of graft-versus-host disease (GvHD), no damage to critical organs, and sustained robustness during inflammatory and antitumor responses (67). Furthermore, the same approach has been applied to generate CAR-iNKT cells targeting solid tumors, including ovarian cancer (70), glioblastoma (71), renal cell carcinoma (RCC) (72), and triple-negative breast cancer (TNBC) (73). These CAR-iNKT cells demonstrate robust persistence and effective tumor infiltration, highlighting their superior antitumor activity. Notably, the incorporation of IL-15 in CAR-NKT cells enhances both their expansion and survival *in vivo*, contributing to improved efficacy while maintaining a favorable safety profile, likely by supporting sustained cytotoxic function without promoting excessive cytokine release.

A recent clinical trial evaluating autologous GD2-targeted CAR-iNKT cells for pediatric neuroblastoma reported a case of hyperleukocytosis (74). This adverse event occurred in the first patient treated at dose level (DL) 5 and represented the initial use of K562-derived artificial antigen-presenting cells (aAPCs) for product restimulation, replacing the previously used autologous peripheral blood mononuclear cells (PBMCs) (7, 8, 74). Notably, none of the twelve patients treated at lower dose levels (DL1–DL4) experienced comparable toxicity. A comprehensive root-cause investigation excluded genetic aberrations of clinical relevance and ruled out insertional mutagenesis-induced clonal expansion. Transcriptomic and functional analyses suggested that the substitution of PBMCs with aAPCs induced a hyperproliferative phenotype and distinct gene expression signatures, potentially driving uncontrolled lymphocyte expansion and the observed toxicity (74). Despite this isolated incident, CAR-iNKT cell products generated using the original PBMC-based expansion protocol have demonstrated favorable safety profiles and antitumor activity in other treated patients. In conclusion, these findings underscore the critical influence of manufacturing variables, particularly restimulation methods, on IL-15-boosted CAR-iNKT cell behavior and clinical outcomes, highlighting the need for rigorous preclinical validation of production modifications to mitigate unforeseen toxicities (74).

### 3.5 IL-18

Interleukin-18 (IL-18) is a pro-inflammatory cytokine belonging to the IL-1 family, with a key role in regulating both the innate and adaptive immune responses. The IL-18 gene is located on chromosome 11 in humans, and its expression is primarily driven by immune cells such as monocytes, macrophages, and neutrophils. IL-18 is central to host defense mechanisms, acting on T helper cells, NK cells, NKT cells, B cells, and dendritic cells to promote the production of IFN-γ, a critical mediator of immune responses (75). IL-18 comprises 12 strands that form three intertwined four-stranded β-sheets, along with a short α-helix and a 310-helix. These β-sheets assemble into a trefoil fold, a structural motif characteristic of the IL-1 family, including IL-1β (76). There are subtle differences in the length of some segments between the strands, such as S3–S4, S4–S5, S7–S8, and S11–S12, which contribute to its specific structural features compared to other IL-1 family members (77).

The IL-18 signaling pathway begins with the formation of a signaling complex through a low-affinity interaction with the IL-18 receptor alpha chain (IL-18Rα). In some cells, IL-18 also binds to the high-affinity IL-18 receptor beta chain (IL-18Rβ). This receptor complex shares similarities with other IL-1 family cytokine signaling pathways, such as those involving IL-1RAcP. The receptor-ligand interaction forms a heterodimer that brings together Toll-IL-1 receptor (TIR) domains, initiating a cascade of intracellular events. This cascade includes the recruitment of the adaptor protein MyD88, followed by the phosphorylation of IRAK family kinases, activation of TRAF6, and the subsequent activation of NFκB. In the presence of other cytokines like IL-12 or IL-15, IL-18 induces IFN-γ production through upregulation of IL-18Rβ, which becomes essential for the full activation of its signaling pathway (75).

IL-18 has emerged as a critical modulator in CAR-engineered cell therapies, where it enhances the proliferation and survival of CAR-T cells. For example, a CD19-targeted CAR-T cell construct incorporating human IL-18 (1928z-hIL18) has been developed and tested in syngeneic mouse models of both hematologic and metastatic solid tumors. IL-18-armored CAR-T cells demonstrated increased secretion of IFN-γ compared to conventional CAR-T cells, along with enhanced *in vivo* expansion and persistence. These IL-18-expressing CAR-T cells exhibited potent anti-tumor effects, not only through direct cytotoxicity but also by modulating the tumor microenvironment. The engineered T cells increased endogenous immune responses, including the expansion of CD8^+^ T cells and NK cells, further amplifying the anti-tumor activity (78).

In summary, IL-18 plays a pivotal role in the immunological enhancement of CAR-T cell therapy, promoting both direct anti-tumor activity and the recruitment of additional immune effectors, thereby improving therapeutic outcomes in cancer immunotherapy.

### 3.6 Mechanistic effects of key cytokines on CAR cell expansion, exhaustion, and cytotoxicity

IL-2 is a potent driver of short-term expansion and effector differentiation in CAR-T cells via IL-2Rβ/γc-dependent JAK/STAT5, PI3K/AKT and RAS/MAPK signaling. These pathways enhance proliferation and effector functions (granzyme and perforin) but also promote terminal differentiation and susceptibility to activation-induced cell death (AICD) under chronic stimulation, accelerating phenotypic exhaustion (PD-1 and TIM-3 upregulation) if uncontrolled. Additionally, systemic IL-2 non-selectively expands Tregs and can exacerbate systemic toxicity such as CRS. Therefore, IL-2’s translational utility in CAR platforms commonly relies on selective delivery strategies, such as orthogonal IL-2/IL-2R pairs, CAR-restricted secretion, or membrane-tethering, to preserve expansion/cytotoxic benefits while limiting bystander activation and exhaustion (79–81).

IL-7 primarily supports homeostatic survival and long-term persistence of naïve and central memory T cell subsets through upregulation of anti-apoptotic proteins such as BCL-2 and maintenance of mitochondrial fitness via STAT5 and PI3K signaling. Unlike IL-2, IL-7 promotes a less differentiated, memory-biased phenotype that is more resistant to exhaustion and retains proliferative capacity upon antigen re-encounter, thereby enhancing durability rather than immediate terminal effector function. Consequently, IL-7 is attractive for engineering CAR constructs aimed at improving persistence and recall responses with a reduced risk of AICD or Treg expansion (82, 83).

IL-10 is classically immunoregulatory, signaling via JAK1/TYK2 and STAT3 to suppress pro-inflammatory pathways including NF-κB and MAPK. Paradoxically, within CAR platforms IL-10 expression has been reported to preserve mitochondrial integrity, reduce chronic inflammatory signaling and thereby limit exhaustion, which can indirectly sustain cytotoxic function over time. In engineered CAR-T cells IL-10 may therefore act to restrain deleterious hyperactivation while maintaining metabolic fitness and effector persistence; however, its anti-inflammatory nature necessitates careful titration since excessive IL-10 expression can blunt acute effector cytokine responses (84, 85).

IL-12 potently drives Th1 polarization and IFN-γ production via STAT4 and promotes NK and CD8^+^ T cell cytotoxic programs, increasing per-cell killing capacity and pro-inflammatory cytokine output. This strong activation enhances tumoricidal activity and can recruit endogenous effectors, but its potent systemic inflammatory signature also creates substantial toxicity risk such as CRS. Chronic IL-12 exposure can lead to exhaustion from sustained IFN-γ and TNF signaling; therefore, local or inducible IL-12 expression is the preferred engineering strategy to harness cytotoxic benefits while minimizing systemic inflammation and exhaustion (55, 86, 87).

IL-15 signals through IL-15Rα/IL-2Rβγc and robustly supports proliferation and survival of memory-phenotype CD8^+^ T cells and NK cells via STAT5 and PI3K/AKT. Unlike IL-2, IL-15 preferentially fosters memory and stem-like phenotypes that resist exhaustion and sustain long-term cytotoxic potential, increasing *in vivo* persistence and repeat-response capability. However, constitutive systemic IL-15 can provoke excessive immune activation and severe CRS; engineering solutions that have shown promise include membrane-bound IL-15, inducible expression, or tethering to the CAR to limit exposure to the tumor locale (23, 59, 60, 88).

IL-18 synergizes with IL-12/IL-15 to amplify IFN-γ production and NK/T cell cytotoxicity via MyD88-dependent signaling cascades (TRAF6, NF-κB and AP-1). IL-18 expression in CAR cells enhances proliferation, recruits and activates bystander immune populations, and remodels the tumor microenvironment toward a pro-inflammatory state. Because IL-18 primarily amplifies effector cytokine production rather than directly enforcing long-term memory programs, its prolonged or systemic presence can accelerate inflammation-associated exhaustion unless tightly regulated; tumor-localized or CAR-stimulated IL-18 release optimizes antitumor synergy while reducing systemic risks (89–91).

IL-21 signaling through STAT3 and STAT1 supports cytotoxic differentiation with features distinct from IL-2. IL-21 promotes expansion of less differentiated effector cells with high proliferative potential and enhances expression of cytotoxic mediators (granzyme B and perforin) while preserving a stem-like program that is relatively resistant to terminal exhaustion. IL-21 also augments B cell and helper T cell functions, so its use in CAR designs may improve persistence and antitumor potency without the severe Treg expansion associated with IL-2. Controlled, CAR-restricted IL-21 delivery can therefore increase cytotoxicity and durability with an acceptable safety profile (92, 93).

## 4 Other engineering strategies to improve efficacy and safety CAR cell therapy

CAR-engineered cell therapy has achieved significant success in the treatment of specific hematologic malignancies and solid tumors. However, challenges such as tumor heterogeneity, immune evasion, and potential toxicity highlight the need for continued advancements in CAR design (**Figure 1**) (94–99). We then explore several promising strategies to overcome these hurdles, including the use of checkpoint inhibitors, chemokine receptor modifications to enhance tumor infiltration, and the incorporation of suicide genes to improve safety.

### 4.1 Checkpoint blockade

Checkpoint inhibitors, such as anti-PD-1 antibodies, have transformed cancer therapy by enabling the immune system to more effectively combat tumors. PD-1 is an inhibitory receptor found on T cells that, when engaged by its ligands, PD-L1 or PD-L2, suppresses T cell activation and promotes immune tolerance. In the context of CAR-T therapy, PD-1 expression on CAR-T cells can lead to T cell exhaustion and diminished therapeutic efficacy (100).

To address this issue, researchers have explored engineering CAR-T cells to express anti-PD-1 receptors. This modification blocks the PD-1 signaling pathway, allowing for enhanced T cell activation and persistence within the tumor microenvironment. Studies have shown that CAR-T cells engineered to express anti-PD-1 exhibit improved anti-tumor activity and prolonged survival in preclinical models. This strategy not only boosts CAR-T cell efficacy but also tackles the problem of T cell exhaustion, which is a major barrier to sustained therapeutic response (101).

The PD-1 pathway plays a critical role in regulating T cell activation and maintaining immune tolerance. As part of the B7/CD28 family of co-stimulatory receptors, PD-1 engagement with its ligands, PD-L1 and PD-L2, suppresses T cell proliferation and the production of key cytokines like IL-2 and IFN-γ. This inhibitory mechanism is particularly significant in chronic infections and cancer, where prolonged TCR stimulation leads to T cell exhaustion, resulting in reduced effector function.

In CAR-T cell therapy, the expression of PD-1 on T cells can severely impede their effectiveness, as tumor cells often express PD-L1, leading to decreased CAR-T cell activation and survival. By engineering CAR-T cells to block the PD-1 interaction, T cell function can be restored, thereby enhancing the anti-tumor response. The differential expression of PD-1 ligands is also significant, as PD-L1 is broadly expressed on various tissues, including tumor cells, while PD-L2 is more restricted to dendritic cells and monocytes. This distinction has therapeutic implications, as targeting PD-1 or PD-L1 could result in different biological outcomes, with PD-L1 blockade preserving PD-L2 interactions, potentially maintaining some degree of immune regulation (102–105).

Similarly, CTLA-4 (Cytotoxic T-Lymphocyte Associated Protein 4) is another pivotal immune checkpoint that regulates T cell activation during the priming phase. CTLA-4 competes with CD28 for binding to B7-1 (CD80) and B7-2 (CD86) on APCs. While CD28 engagement provides co-stimulatory signals for T cell activation, CTLA-4 binding delivers an inhibitory signal that prevents T cell activation, particularly during high levels of TCR stimulation, leading to immune tolerance and anergy (106).

Targeting CTLA-4 in CAR-T cell therapy provides an opportunity to enhance T cell activation and proliferation. Inhibiting CTLA-4 allows CAR-T cells to produce higher levels of IL-2 and sustain their survival, thereby increasing their efficacy against tumors. Additionally, CTLA-4 is constitutively expressed on regulatory T cells (Tregs), which suppress effector T cell responses. Modulating CTLA-4 in Tregs may reduce this suppression and further enhance anti-tumor immunity. Research into CTLA-4 blockade has shown that it increases the diversity and activation of T cell clones, contributing to a more robust immune response. Combining CTLA-4 and PD-1 pathway blockades has demonstrated potential synergistic effects, as CTLA-4 inhibition enhances T cell priming, while PD-1 blockade restores the function of exhausted T cells. This combined approach is being actively investigated for its potential to improve outcomes in CAR-T cell therapies and other forms of immunotherapy (106).

### 4.2 Chemokine receptor modifications

The TME presents significant challenges for CAR-T cell therapies, characterized by limited homing signals, physical barriers, and the presence of immunosuppressive factors that impede effective tumor infiltration and activity. Chemokine receptors are critical in guiding immune cells to sites of inflammation and tumors. By engineering CAR-T cells to express specific chemokine receptors, their ability to migrate and infiltrate tumor sites can be significantly enhanced, improving their therapeutic efficacy (107–111).

For example, CAR-T cells engineered to express chemokine receptors such as CXCR1, CXCR2, or CCR2 demonstrate enhanced migration toward tumor sites that secrete corresponding chemokines, such as CXCL8 for CXCR1/2 and CCL2 for CCR2. This approach facilitates the efficient trafficking of CAR-T cells to tumor tissues, improving their ability to accumulate in the TME and overcome the barriers posed by immunosuppressive factors. Not only does this strategy increase the likelihood of CAR-T cells reaching and persisting at the tumor site, but it also enhances their capacity to engage and kill tumor cells, leading to improved anti-tumor responses (112).

Moreover, chemokine receptor modifications can be tailored to the specific chemokine profile of different tumor types, allowing for a more personalized and targeted approach. For instance, breast cancer or glioblastoma, which are known to produce high levels of CCL2, may benefit from CAR-T cells engineered to express CCR2, increasing the therapeutic potential of the treatment. This adaptability ensures that CAR-T cell therapies can be fine-tuned to the unique characteristics of the patient’s tumor microenvironment, optimizing their efficacy across a range of solid tumors. Additionally, the combination of chemokine receptor engineering with other strategies, such as immune checkpoint inhibition, can further enhance the functionality of CAR-T cells within the suppressive TME, offering a more comprehensive approach to overcoming the limitations of CAR-T cell therapy in solid tumors.

### 4.3 Incorporation of suicide genes

While CAR-T therapy has shown great promise in treating certain cancers, the risk of severe adverse effects, such as CRS and neurotoxicity, remains a major safety concern (70, 71, 113–115). One innovative approach to mitigating these risks is the incorporation of suicide genes into CAR-T cells, which can be activated to induce apoptosis in the CAR-T cells upon administration of a specific drug or in response to a predefined stimulus.

For instance, the use of the herpes simplex virus thymidine kinase (HSV-TK) gene allows for the selective elimination of CAR-T cells through the administration of ganciclovir (116–118). This strategy acts as a safety switch, enabling clinicians to reduce or eliminate CAR-T cells in cases of severe side effects, such as uncontrolled CRS or neurotoxicity. Similarly, the inducible caspase 9 (iCasp9) system provides a more refined method for controlling CAR-T cells. In this system, apoptosis can be triggered in response to the administration of a small molecule, such as AP1903, allowing for rapid and controlled depletion of CAR-T cells when adverse events occur (119).

These suicide gene systems offer an additional layer of safety in CAR-T cell therapy, giving clinicians greater control over the treatment process and reducing the risk of potentially life-threatening complications. As a result, the incorporation of suicide genes in CAR-T therapies is an important step toward improving the overall safety profile of these powerful cancer treatments. Importantly, however, severe CRS is far less common in CAR-NK and CAR-NKT cell–based therapies. Unlike CAR-T cells, NK and NKT cells possess intrinsic regulatory programs, such as limited proliferative bursts, reduced IL-6 production, and the ability to undergo activation-induced apoptosis, that naturally temper excessive inflammatory responses (11, 120–122). Clinical studies have also shown that CAR-NK and CAR-NKT cells can mediate strong antitumor activity with minimal CRS or neurotoxicity, even in dose-escalation settings (7, 123). This inherent safety advantage has shifted interest toward NK- and NKT-based platforms as next-generation cellular therapies, where the need for externally controlled safety switches may be reduced. Together, these developments highlight how suicide gene technologies and alternative immune effector cell types can complement one another, collectively advancing the safety and translational potential of engineered cell therapies.

## 5 Conclusion

In conclusion, the application of cytokine engineering into CAR cell therapy delivers a promising approach to enhancing the efficacy of immunotherapy, particularly in the difficult treatment of solid tumors (124). This paper elucidates the multifaceted roles of key cytokines, such as IL-2, IL-7, IL-10, IL-12, IL-15, IL-18, and IL-21, in modulating T cell behavior, improving persistence, and overcoming the immunosuppressive barriers of the tumor microenvironment (**Table 1**) (23, 89, 125, 126). By harnessing the unique biological properties of these cytokines, researchers have demonstrated significant improvements in CAR-T cell proliferation, functionality, and overall anti-tumor responses.

In addition to the cytokines discussed above, IL-33 has emerged as another promising candidate for cytokine engineering. Recent studies demonstrate that CAR-T cells armored with Superkine IL-2 and IL-33 can remodel the tumor microenvironment, enhance antitumor immunity, and suppress the growth of multiple solid tumors, with evidence suggesting that this combination may also mitigate antigen-loss–driven resistance (43, 127). These findings underscore the potential relevance of IL-33–based cytokine strategies in the development of next-generation engineered cell therapies.

The selection of cytokines for CAR cell engineering is based on their complementary immunological functions, capacity to modulate T and NK cell behavior, and safety profiles within the TME. Cytokines such as IL-2 and IL-12 are traditionally incorporated to maximize effector activation and cytotoxicity, as they strongly enhance proliferation, IFN-γ production, and direct tumor lysis. However, due to their association with terminal differentiation, T-cell exhaustion, and systemic inflammation, their use now favors localized or inducible expression to retain potency while mitigating toxicity. In contrast, IL-7, IL-15, and IL-21 are prioritized for promoting long-lived, memory-like phenotypes that maintain metabolic resilience and sustained *in vivo* persistence, which are attributes critical for overcoming relapse and immune evasion in solid tumors. IL-15, in particular, supports homeostatic proliferation of memory T and NK cells, whereas IL-21 balances effector differentiation with self-renewal capacity. Meanwhile, IL-10 and IL-18 are selected for their regulatory and immunomodulatory roles: IL-10 tempers chronic inflammatory signaling to preserve functional fitness, and IL-18 recruits innate immune subsets, amplifying endogenous antitumor immunity and remodeling the TME. Thus, the rationale for cytokine selection is to create a coordinated network that enhances proliferation and cytotoxic strength (IL-2, IL-12, IL-18) while simultaneously sustaining persistence and minimizing exhaustion (IL-7, IL-15, IL-21, IL-10). Strategic combinations of these cytokines allow for the customization of CAR constructs that achieve an optimal balance between potency, persistence, and safety.

The promising results from preclinical and clinical studies underscore the potential of cytokine-enhanced CAR therapies to not only augment the therapeutic impact against hematologic malignancies but also to extend their applicability to solid tumors, where traditional CAR-T cell approaches have struggled. Furthermore, the exploration of innovative CAR designs, including the incorporation of suicide genes and checkpoint inhibitors, alongside cytokine engineering, offers a comprehensive strategy to mitigate adverse effects and enhance the safety profile of these therapies.

Although cytokine-engineered CAR-T cell therapy represents a promising frontier in enhancing efficacy and persistence, its potential toxicities and safety concerns warrant careful consideration. For instance, IL-2 remains a powerful stimulator of CAR-T expansion but poses significant clinical toxicity risks when administered in its untargeted form, largely due to systemic immune activation and vascular permeability effects. In contrast, IL-15 and CAR-restricted cytokine engineering strategies offer more favorable translational prospects by promoting T-cell persistence and memory formation while reducing bystander immune activation.

Current translational efforts emphasize achieving selective cytokine signaling through several approaches: (i) engineering CAR-T cells to secrete locally restricted cytokines such as membrane-tethered or inducible IL-15; (ii) developing cytokine muteins or orthogonal IL-2/IL-2R systems that preferentially signal within engineered cells; and (iii) minimizing systemic cytokine exposure through temporally controlled dosing regimens. These strategies aim to retain the proliferative and survival benefits of cytokine support while mitigating systemic inflammatory toxicity. Rigorous preclinical evaluation and early-phase clinical trials with comprehensive immune monitoring are therefore essential prerequisites for the safe and effective clinical deployment of cytokine-augmented CAR-T cell therapies.

Next-generation cytokine engineering strategies are increasingly leveraging gene-editing and synthetic biology tools to achieve precise and dynamic control of cytokine expression in CAR-modified immune cells. CRISPR-based genetic circuits now enable programmable cytokine release in response to defined environmental or intracellular cues, such as tumor antigen engagement or hypoxia, thereby restricting cytokine activity to the tumor site while minimizing systemic toxicity (128–132). These synthetic circuits can be integrated into CAR constructs to modulate cytokine output—such as IL-2, IL-12, or IL-18—only upon CAR signaling, reducing off-target inflammation. Inducible cytokine release systems represent another key innovation, using drug- or light-sensitive promoters to allow reversible cytokine activation (124, 133, 134). Such designs permit clinicians to fine-tune cytokine support during therapy, mitigating toxicity risks like cytokine release syndrome while sustaining CAR persistence when needed. Moreover, synthetic biology frameworks now support the design of modular “logic-gated” cytokine circuits that combine Boolean (AND/NOT) signaling logic to enhance selectivity. For instance, dual-antigen–sensing CAR platforms can trigger IL-15 or IL-21 secretion only in tumor-restricted contexts, offering an added safety layer (135, 136). Collectively, these tools redefine the therapeutic landscape by merging immunoengineering with programmable control systems, paving the way for adaptive, precision-controlled CAR therapies with improved efficacy and safety.

As we advance, continued research into the optimal combinations and delivery mechanisms for cytokine-modulated CAR therapies will be essential (137–142). The insights gained from these studies will pave the way for the development of more effective and personalized cancer treatments, with the goal of improving patient outcomes and expanding the horizons of CAR cell therapy in immunotherapy. The future of cancer treatment lies in the strategic engineering of immune cells, and cytokine engineering stands at the forefront of this evolution, promising to revolutionize the landscape of cancer treatment.
